# Testing a Consumer Wearables Program to Promote the Use of Positive Airway Pressure Therapy in Patients With Obstructive Sleep Apnea: Protocol for a Pilot Randomized Controlled Trial

**DOI:** 10.2196/60769

**Published:** 2024-09-19

**Authors:** Selene Mak, Garrett Ash, Li-Jung Liang, Erin Der-McLeod, Sara Ghadimi, Anjali Kewalramani, Saadia Naeem, Michelle Zeidler, Constance Fung

**Affiliations:** 1 Center for the Study of Healthcare Innovation, Implementation and Policy (CSHIIP) VA Greater Los Angeles Healthcare System Los Angeles, CA United States; 2 Section of General Internal Medicine Yale School of Medicine New Haven, CT United States; 3 Section of Biomedical Informatics and Data Science Yale School of Medicine New Haven, CT United States; 4 Center for Pain, Research, Informatics, Medical Comorbidities and Education Center (PRIME) VA Connecticut Health System West Haven, CT United States; 5 Department of Medicine David Geffen School of Medicine at UCLA Los Angeles, CA United States; 6 Geriatric Research Education and Clinical Center North Hills, CA United States; 7 Sleep Center VA Greater Los Angeles Healthcare System Los Angeles, CA United States

**Keywords:** sleep apnea, consumer wearables, adherence, self-management, mobile phone

## Abstract

**Background:**

Although positive airway pressure (PAP) therapy is considered first-line treatment for obstructive sleep apnea (OSA), nonadherence is common. Numerous factors influence PAP use, including a belief that the therapy is important and effective. In theory, providing information to patients about their blood oxygen levels during sleep (which may be low when PAP is not used), juxtaposed to information about their PAP use, may influence a patient’s beliefs about therapy and increase PAP use. With the advent of consumer wearable smartwatches’ blood oxygen saturation monitoring capability (and the existing routine availability of PAP use data transmitted via modem to clinical dashboards), there is an opportunity to provide this combination of information to patients.

**Objective:**

This study aims to test the feasibility, acceptability, and preliminary efficacy of the Chronic Care Management With Wearable Devices in Patients Prescribed Positive Airway Pressure Therapy (mPAP), a program that augments current PAP therapy data with consumer-grade wearable device to promote self-management of PAP therapy for OSA in a pilot randomized waitlist-controlled clinical trial.

**Methods:**

This is a single-blinded randomized controlled trial. We will randomize 50 individuals with a history of OSA, who receive care from a Department of Veterans Affairs medical center in the Los Angeles area and are nonadherent to prescribed PAP therapy, into either an immediate intervention group or a waitlist control group. During a 28-day intervention, the participants will wear a study-provided consumer wearable device and complete a weekly survey about their OSA symptoms. A report that summarizes consumer wearable–provided oxygen saturation values, PAP use derived from modem data, and patient-reported OSA symptoms will be prepared weekly and shared with the patient. The immediate intervention group will begin intervention immediately after randomization (T1). Assessments will occur at week 5 (T3; 1 week after treatment for the immediate intervention group and repeat baseline for the waitlist control group) and week 11 (T5; follow-up for the immediate intervention group and 1 week after treatment for the waitlist control group). The primary outcome will be the change in 7-day PAP adherence (average minutes per night) from T1 to T3. The primary analysis will be a comparison of the primary outcome between the immediate intervention and the waitlist control groups (intention-to-treat design), using a 2-sample, 2-sided *t* test on change scores (unadjusted).

**Results:**

Recruitment began in October 2023. Data analysis is expected to begin in October 2024 when all follow-ups are complete, and a manuscript summarizing trial results will be submitted following completion of data analysis.

**Conclusions:**

Findings from the study may provide additional insights on how patients with OSA might use patient-generated health data collected by consumer wearables to inform self-management of OSA and possibly increase their use of PAP therapy.

**Trial Registration:**

ClinicalTrials.gov NCT06039865; https://clinicaltrials.gov/study/NCT06039865

**International Registered Report Identifier (IRRID):**

DERR1-10.2196/60769

## Introduction

### Background

Obstructive sleep apnea (OSA) is prevalent and affects more veterans who obtain care in Veterans Health Administration facilities than any other sleep disorder (20%) [1]. Characterized by repetitive blockage in the airway during sleep that causes breathing to stop or get very shallow and that may cause transient hypoxemia, OSA disrupts sleep and contributes to symptoms of excessive daytime sleepiness; decreased quality of life; and increased risk of health conditions such as depression, cardiovascular disease, erectile dysfunction, and dementia [1]. OSA is also associated with work disability [2] and increased risk of motor vehicle crashes [1].

OSA is a chronic disease that requires long-term management [3]. Positive airway pressure (PAP) devices, which deliver air under pressure into the airway to stent the airways open, are considered the gold standard treatment for OSA. The success of this treatment is quantified by hourly and nightly adherence plus changes to symptoms (eg, daytime sleepiness) and reduction in the number of times per hour airflow ceases or is reduced, as well as improvement in sleep-related oxygen levels [4,5]. Adherence to treatment can improve sleep quality, reduce the risk of OSA-related comorbidities, and improve quality of life [6]. However, low PAP adherence is a common issue [7].

Among a number of important factors that influence whether a patient uses prescribed therapy is the belief that the therapy is important and effective [2]. Through clinical experience and a prior project that have focused on patients’ decision-making about their OSA and treatment, we found that providing information to patients about their blood oxygen levels (eg, lowest blood oxygen level) during sleep is very powerful as a tool for engaging them in their OSA management. Studies have shown that data visualizations may be associated with positive effects on decision-making due to the decreased cognitive and intellectual burden of interpreting data [8,9]. With the advent of consumer wearable smartwatches’ (hereafter smartwatch) blood oxygen saturation (SpO_2_) monitoring capability, there is both an opportunity and an impending need to optimize the manner in which this information is used in patients’ daily lives and in routine sleep clinic care so that the information enhances patients’ experience with care.

Increased affordability and relative convenience of putting a device on the wrist to continuously collect physiological data (eg, steps and heart rate) have popularized smartwatches for everyday use. Manufacturers have also developed proprietary algorithms using data collected with various sensors to estimate sleep-related metrics such as sleep time, wake time, and sleep stages. The result is a great deal of data available to the consumer that may appear medically relevant, particularly against the backdrop of beautifully designed interfaces and eye-catching graphics. However, these watches are designed to be consumer-facing products, such that the data presented should not be used to replace clinical screening or diagnosis of sleep disorders such as OSA, even if they have features to detect signs of moderate to severe OSA [10]. While there is limited evidence to the reliability [11] and validity [12-14] of the data for clinical purposes, there may be benefits to tracking health behaviors for self-monitoring purposes [15].

Newer models of consumer-facing smartwatches (eg, Fitbit) are providing SpO_2_ measurements in minute-by-minute intervals [16]. Although these wearables are not intended for medical purposes such as diagnosing OSA, they are currently used by patients for self-monitoring oxygen levels, which can drop to low levels in some patients when they are not using PAP therapy. Theoretically, patients who use their PAP therapy inconsistently may have patterns in consumer wearable data that correlate with PAP use. Given the high prevalence of OSA and the growing use of smartwatches, inevitably a subset of patients with OSA will use smartwatches for self-monitoring their oxygen levels during sleep and will present to their sleep clinicians with questions about their wearable oxygen levels. A conundrum is how best to incorporate the vast amount of sleep data from consumer technology into the chronic care management of patients with sleep disorders such as OSA.

Although the PAP devices include airflow sensors that can provide physiologic data feedback [17], additional feedback about oxygen levels may influence behavior. The proposed research study will incorporate the use of a wearable collecting SpO_2_ data during sleep and provide a weekly report that will not only depict the physiological consequences of nonadherence as a timely behavioral reinforcement strategy, but also highlight day-to-day relationships of adherence and possible barriers (eg, lower adherence on nights of greater pain). In conjunction with PAP device data and patient-reported outcomes (eg, daytime sleepiness and alertness), the wearable data represent an opportunity to augment current strategies for increasing use of PAP therapy.

The Health Beliefs Model provides a framework for incorporating wearable data (specifically oxygen saturation data) in a PAP therapy program [18]. The model suggests that the desire to get well if already ill (eg, improve OSA) and the belief that a specific health action will cure illness, together, influence health behavior (eg, use PAP for OSA), and the patient’s course of action often depends upon the patient’s perceptions of the benefits and barriers related to that health behavior (eg, PAP use). The perceptions influencing PAP use may include (1) perceived susceptibility—if levels of oxygen saturation collected during sleep are intermittently low, (2) perceived severity—if oxygen levels are lower than normal values, and (3) perceived benefits—if oxygen levels during sleep are different on days PAP used versus not used. Cues to action (eg, PAP use) may be feedback showing wearable oxygen saturation data combined with PAP use and patient-reported outcomes.

The goal of the Chronic Care Management With Wearable Devices in Patients Prescribed Positive Airway Pressure Therapy (mPAP) project is to assess the impact and acceptability of augmenting current PAP therapy data with consumer-grade wearable device data to promote self-management of PAP therapy for OSA. To isolate the impact of the augmentation by the wearable device, the comparator intervention is receiving current PAP therapy data while on a waitlist to add the wearable device data.

### Study Aims and Hypotheses

The specific aims and hypotheses are as follows:

- Specific aim 1: to measure the effect of the consumer grade wearable–augmented program on PAP use.
  - Primary hypothesis: compared to participants in the waitlist control group, participants in the immediate intervention group will have greater improvement in PAP adherence (change in average minutes per night from baseline [T1] to posttreatment assessment [T3]).
  - Secondary hypothesis: PAP adherence (average minutes per night) from delayed baseline (T4) to delayed posttreatment assessment (T5) will improve among participants in the waitlist control group after receiving the delayed wearable intervention.
  - Exploratory hypotheses: the effect of the program will be maintained at T5 for the immediate intervention group.
- Specific aim 2: to assess the acceptability of the program.

## Methods

### Study Design

This study is a randomized parallel 2-group waitlist-controlled trial with 1:1 allocation evaluating the efficacy of a consumer wearable–augmented self-monitoring program on PAP adherence compared to a standard self-monitoring program on PAP adherence among patients with OSA prescribed PAP therapy. In addition, following the primary end point, the control group will crossover to the intervention condition and be compared to their time in the control condition (ie, single-group nonrandomized crossover design). The SPIRIT (Standard Protocol Items: Recommendations for Interventional Trials) 2013 statement was used to report standard protocol items for clinical trials [19].

### Study Site

The study will be conducted at Veterans Affairs Greater Los Angeles Healthcare System in Los Angeles, California.

### Participants: Inclusion and Exclusion Criteria

Inclusion criteria were as follows: aged ≥21 years, prescribed PAP therapy from the sleep center for >1 week, have an active profile in our Center’s ResMed AirView (ResMed) or Respironics Care Orchestrator (Philips Respironics) accounts for >1 week, willing to continue using current PAP device for 28 days continuously, nonadherent to PAP therapy (ie, not using their PAP for 4 or more hours per night on 70% of nights during a consecutive 30-day period) [20], and have a smartphone compatible with the wearable app.

Exclusion criteria were as follows: conditions that may cause nocturnal hypoxemia unrelated to OSA (ie, heart failure, chronic lung disease, and home oxygen use); conditions that may limit individual’s ability to follow study procedures such as wear Fitbit, attempt to use their PAP, participate in interviews with research team, and complete web-based surveys (ie, dementia, active substance use disorder, unstable medical or psychiatric illness, concurrent participation in another clinical trial that would interfere with study procedures, planned nonuse of PAP therapy, and planned surgery or hospitalization during study period that would limit PAP use); planned extensive travel during study period; and history of repeated nonattendance at clinic visits. Textbox 1 provides details on how we will operationalize the exclusion criteria.

**Operationalizing exclusion criteria.**

**Exclusion criteria**

- Conditions that may cause nocturnal hypoxemia unrelated to obstructive sleep apnea
  Heart failure: heart failure diagnosis listed in the electronic health record problem list
  Chronic lung disease: lung conditions that can cause hypoxemia including asthma, chronic obstructive lung disease, and restrictive lung disease
  Home oxygen use: evidence in the electronic health record or patient report of prescription for home oxygen
- Conditions that may interfere with the protocol
  Dementia: dementia reported by patient and/or caregiver or listed in the electronic health record
  Active substance use disorder interfering with the protocol (investigator discretion)
  Unstable medical or psychiatric illness interfering with the protocol (investigator discretion)
  Planned surgery or hospitalization during study period interfering with the protocol (positive airway pressure [PAP] access or investigator discretion)
  Planned extensive travel during study period interrupting electricity, cellular and/or internet service, or the protocol (investigator discretion)
  History of repeated nonattendance at clinic visits
- Refusal to engage in any PAP therapy
  No future intention of resuming PAP use

### Procedures

#### Recruitment and Screening

Recruitment strategies will include (1) mailing letters to patients with active modem accounts, (2) posting flyers or posters in the clinic areas and enclosing flyers with PAP equipment distributed to patients, and (3) notifying sleep center staff about the study. Participants will undergo an initial telephone screen to assess for eligibility and to collect demographic data (age, gender, race, and ethnicity). Research staff will perform a brief medical chart review to verify eligibility and OSA history (diagnosis date, severity [apnea-hypopnea index], and PAP settings [fixed vs auto]). We will verify that the patient has a PAP modem profile on the clinic’s AirView or Care Orchestrator account. The research team will meet weekly to review eligibility requirements for each individual after completing baseline assessment and 1-night oximetry to determine whether the individual is eligible for randomization in the clinical trial.

We increased the number of letters mailed each week to patients meeting the inclusion criteria to help us achieve adequate participant enrollment to reach target sample size. Another strategy used was to present this study to the Greater Los Angeles Veteran Engagement Team (CIN 13-417) in December 2023 for feedback on our recruitment strategy. The Greater Los Angeles Veteran Engagement Team is composed of a diverse team of local veterans whose mission is to partner as active stakeholders with the Greater Los Angeles research community and impart veteran-based knowledge and perspectives on research and other improvement efforts. Some of their suggestions include posting flyers in additional locations with more foot traffic.

#### Assessments

##### Baseline Assessment

Participants in both arms will undergo a baseline assessment (T1 for the immediate intervention group; T3 for the waitlist control group), where current 7-day and 30-day PAP adherence will be collected from modem data. One-night oximetry using a United States Food and Drug Administration–approved wrist-worn pulse oximeter (Nonin) will be conducted. Other measures such as determinants of PAP compliance (ie, self-efficacy, knowledge, stages of change, and decisional balance index) [21], sleep disturbance, sleep quality, daytime function, comorbidities, and health literacy will be collected through an electronic survey using the Qualtrics platform. A baseline assessment appointment with a research team member will be conducted, where the research team member will review the survey and administer any items that were unintentionally left incomplete.

##### Follow-Up Assessment

The immediate intervention group will undergo posttreatment assessment at T3 and follow-up assessment at T5. The waitlist control group will complete the posttreatment assessment at T5. Participants will also undergo an intervention program evaluation upon completion of the intervention (either T3 or T5, depending upon group assignment). At T3 and T5, 7-day and 30-day PAP adherence data will be abstracted from modem data, regardless of whether participants wear the watch or view the reports.

#### Randomization

Eligible participants will be randomized to 1 of the 2 study arms: an immediate intervention and waitlist control, using a 1:1 ratio. Randomization will be performed using a random permuted block design with varying block size generated by the study statistician. Opaque sequentially numbered envelopes were prepared by a researcher who was not involved in any part of our study, thus implementing the random allocation sequence that randomized each successive eligible participant.

#### Blinding

The primary and secondary outcomes at follow-up (ie, T3—week 5 and T5—week 11) will be assessed by research team members who are blinded to study arm assignment. The portion of the assessment covering program evaluation will be administered by an unblinded team member. Participants will not specifically be informed that they are in the immediate versus waitlist group; rather, they will be informed of the expected date of beginning the program.

#### Device Selection

We selected the Fitbit Charge 5 (Google Inc), a smartwatch with a sensor that can sample SpO_2_ in minute-by-minute intervals. There were three considerations:

1. Whether the device had the sensor capable of collecting SpO_2_ data. The Fitbit Charge 5 has “red and infrared light sensors on the back of the device. [These] sensors shine red and infrared light onto skin and blood vessels and use the reflected light that bounces back to estimate how much oxygen is in the blood. Machine learning is used to estimate the SpO_2_ value and to ensure a high confidence level in the blood oxygen reading” [22].
2. Whether we would get data suitable for our purposes. Unlike manufacturers that provide a 15- or 30-minute average SpO_2_ value, or present “high” or “low” variation in oxygen, none of which are useful for self-management of OSA, the Fitbit Charge 5 sampled SpO_2_ in minute-by-minute intervals. For purposes of self-monitoring SpO_2_ while sleeping, it is critical that the data are sampled at sufficiently small intervals to allow for appropriate characterization of abnormal oxygen saturation (eg, SpO_2_<93%).
3. Whether participants would wear the device night after night. Although there are other devices, such as an oximeter, that could provide similar data, we chose a smartwatch because it is more likely that an individual would keep wearing a smartwatch over putting on the oximeter sensor night after night. As participants get to keep the smartwatch after the trial, we hope that many will continue to use the device for its convenience and myriad of features, tracking SpO_2_ or otherwise.

#### Intervention

##### Immediate Intervention: Consumer-Grade Wearable-Augmented PAP Adherence Intervention Program

Immediately following randomization (day 0), each eligible participant assigned to the immediate intervention group will receive a consumer-grade wearable , which records minute-by-minute SpO_2_ data (Fitbit Charge 5), on day 1, along with instructions on how to use it. They will be asked to wear the wearable nightly, even if not using treatment device. Participants will be oriented to using their wearable for the self-monitoring program, including a feature that includes viewing their personal reports on demand. Participants who are not available for an in-person visit will be sent the device and scheduled for a telehealth visit with the research team. Participants will share data with project team via a third-party platform (Fitabase) and will be sent a weekly survey about OSA-related symptoms. The weekly survey about each 7-day period will be sent the morning of day 7, 14, 21, and 28. An automated reminder will be sent after 12 hours if the participant has not completed the survey. We will track the percentage of surveys completed as part of monitoring adherence to intervention protocols.

Data shared by participants will be used to generate customized reports that emphasize the amount of time participant had SpO_2_ values in the various oxygen range categories (eg, minutes spent >92% SpO_2_), PAP use modem data, and responses from the participant about their OSA-related symptoms. The juxtaposition of these 3 different types of information (SpO_2_, PAP use, and symptoms) is intended to help participants identify patterns of association between PAP use and objective (SpO_2_) and subjective (symptoms) data. Within 1 to 2 days of the first report delivery (or scheduled for delivery) to the participant, the participant will meet with a member of the research team, who will review the report and the meaning of the information presented using a structured session guide. The participants will continue to complete an OSA-related symptoms survey weekly until day 28 so that weekly customized reports can be delivered to the participant (also using the Qualtrics platform). The report is presented as an embedded image in the Qualtrics survey that participants can view and download.

The allocated intervention may be modified or discontinued at the participant’s request or by the study team in cases where the intervention (ie, charts) causes severe anxiety or distress or PAP treatment causes harms (eg, dermatitis that is persistent despite changing interfaces). Permitted concomitant care and interventions include those recommended and provided by the participant’s provider within the sleep center (eg, mask fitting, psychoeducation, and PAP desensitization).

##### Waitlist Control

Participants assigned to waitlist control will receive usual care from the sleep center, which includes clinic visit every 6 to 12 months on average, with access to durable medical equipment such as PAP interfaces on an as-needed basis. Participants will also have walk-in access to sleep technologists and respiratory therapists who can answer questions about PAP device issues and can fit patients with a new PAP mask. Each device can be paired with a self-monitoring app by the manufacturer so that patients can monitor adherence (eg, myAir [ResMed] and DreamMapper [Philips Respironics Inc]), and patients also receive feedback about their PAP use on the device display. These participants will receive instructions on the wearable at time point T4 and be offered the same intervention as the immediate group until T5 (28 days).

#### Program Evaluation

We will conduct a semistructured interview with participants at the completion of the program to obtain feedback from participants about the program and to elucidate opportunities to improve the trial design. Research staff with experience conducting program evaluation interviews will conduct these interviews using a semistructured interview guide with questions such as “What did you think about the reports?” or “What did you think about using a Fitbit to collect pulse oximetry data while you slept?” The staff will ask the participants how many of the reports they viewed and if they had any technical difficulties opening or viewing them. Those who choose not to view any reports will still be interviewed for their impressions of the process and concept. We will record and transcribe interviews for analysis.

#### Participant Timeline

Participants are enrolled on a rolling basis. After baseline assessment (T1), participants are randomized into either the immediate intervention group or the waitlist control group. Participants in the immediate intervention group start the 28-day program immediately (T2). After 7 days, at T3, the participants in the immediate intervention group undergo posttreatment assessment, and participants in the waitlist control group complete delayed baseline assessment. At T4, 7 days after T3, waitlist control participants start the intervention for 28 days. At T5, 7 days after intervention completion, participants in the waitlist control group undergo posttreatment assessment and participants in the immediate intervention group complete follow-up assessment.

### Measures

#### Sample Characteristics

Participants will be asked to provide demographic characteristics, such as age, gender, race, ethnicity, marital status, and education level. We will also abstract from the medical record OSA history (severity and PAP settings). Comorbidities will be summarized by the Self-Administered Comorbidity questionnaire (convergent validity with Charlson Comorbidity index *r*=0.55) [23]. Participants' overnight oxygen desaturation, based on clinical overnight oximetry, will be summarized by the amount of time spent at various oxygen saturation levels.

#### Primary Outcome Measure

The primary outcome measure will be the change in PAP adherence (average minutes per night over a 7-day period) from T1 to T3. PAP adherence, defined as the use of the device for 4 or more hours per night on 70% of nights during a consecutive 30-day period [20], is measured continuously and is recorded by the participant’s PAP machine and transmitted to the participant’s PAP account (eg, AirView and Care Orchestrator) through a cellular modem that is embedded in the device [24]. PAP devices can distinguish between periods when the mask is on versus off the face (by detecting flow).

#### Secondary Outcome Measures

Additional PAP adherence metrics will be abstracted from the participant’s PAP modem report: Percentage of days used for 4 or more hours, number of days used out of 7 days, and average residual apnea-hypopnea index on therapy.

Participant acceptability will be assessed [25]. We will review the data and evaluate the proportion of nights participants used the wearable overall and while on treatment to ascertain feasibility and acceptability of using a wearable to collect SpO_2_ data. We will further assess intervention feasibility, acceptability, and appropriateness with items developed by Weiner et al [25]. The postprogram survey will be followed by an interview with patients about their experience using the wearable with treatment, including opinions about the wearable program and intention to continue using the wearable.

#### Other Measures

Sleep disturbance and sleep-related impairment will be measured by the 8-item Patient-Reported Outcomes Measurement Information System (PROMIS) Sleep Disturbance and 8-item Sleep-Related Impairment [26]. Scores are presented as *t* scores, with an average of 50 and SD of 10. Scores >60 are considered elevated. Internal consistency has previously been very high (Cronbach α>0.94) [27].

Hypersomnolence will be assessed with the 8-item Epworth Sleepiness Scale [28]. Score ranges from 0 to 24 (internal consistency Cronbach α=0.82) [29].

Determinants of PAP compliance subscales (self-efficacy, 5 items; knowledge, 12 items) and transtheoretical model (decision balance, 11 items; stages of change, 1 item), chosen from constructs of social cognitive theory, will measure factors that are commonly associated with PAP use (subscale Cronbach α=0.66-0.93) [21].

Graph literacy will be evaluated using a 4-item Short Graph Literacy Scale [30] that presents data in the form of a chart and queries the respondent’s comprehension, with the number of correct answers out of 4 indicating graph literacy. This short scale has convergent validity with both the Long Graph Literacy Scale (*r*=0.90) and a detailed verbal query of how well the participant comprehended the graph (*r*=0.44).

The impact of OSA on biopsychosocial aspects (eg, mood and/or psychosocial outcomes) will be assessed using the Symptoms, Tiredness, Alertness, Mood, and Psychosocial questionnaire [31]. It is a 12-item questionnaire that is scored on a 6-point scale (internal consistency Cronbach α=0.91).

### Statistical Analysis

An intention-to-treat approach will be used. Demographic and clinical characteristics, as well as all outcome measures at baseline, will be summarized using descriptive statistics by study arm to assess baseline comparability. If the distribution of the continuous variable is skewed, an appropriate variable transformation will be made before the comparison. Multiple imputation will be implemented to account for missing data using software for regression models developed by Belin et al [32], Liu et al [33], and Schafer [34].

Changes in sleep-related outcome measures will be analyzed to assess the “success” (effect) of the wearable intervention. The primary sleep-related outcome is change in average minutes per night of PAP use from T1 to T3. Our primary hypothesis is that the proposed intervention program will improve PAP adherence among participants who receive the program immediately compared to a waitlist control group. The primary analysis will be a comparison of mean change in PAP use between the immediate intervention group and the waitlist control group using a 2-sample, 2-sided *t* test (unadjusted). Next, we will construct an analysis of covariance regression model that includes baseline PAP adherence score, study arm (intervention vs control), demographic characteristics (eg, age), and preselected clinical characteristics (eg, determinants of PAP compliance score, OSA severity, and PAP setting) to examine whether the effect of the wearable intervention on PAP adherence, where the change score is the primary outcome, is significant. Because this is a single-site pilot, we have no plans to conduct any interim analyses that would be used to determine termination of trial.

Secondary analyses will compare secondary outcomes, such as the percentage of days used for ≥4 hours out of 28 days and the percentage of days used out of 28 days, between study arms. These comparisons will use generalized linear mixed-effects models with appropriate link functions, such as logit for binary outcomes and log for count outcomes. Each regression model will include the fixed effects of interest (study arm, time, and a 2-way study arm-by-time interaction term) and participant-level random effects to account for dependency of repeated observation within participants. Similar to the primary analysis, adjusted analyses of secondary outcomes with preselected factors will be performed. In addition, a generalized linear mixed-effects model will be used to compare change scores before and after the wearable intervention within the waitlist control group to estimate whether the difference in change scores before and after is significant (secondary hypothesis). This analysis will include the PAP adherence measurements from T1 to T5 for all the waitlist control participants. A similar modeling approach will be used to evaluate whether the effect of the 28-day wearable intervention is maintained at the end of the study (exploratory analysis).

Participant acceptability will be measured and analyzed to assess acceptability. We will summarize acceptability measures (frequency, mean, etc). For interview data, we will summarize interview content thematically using Veterans Affairs (VA)–approved qualitative data analysis software (eg, ATLAS.ti) and identify ways to improve the protocol for a future full-scale trial.

### Power and Sample Size Considerations

A sample size of 25 participants per study arm will provide at least 80% power to detect a standardized effect size (Cohen *d*) of 0.72 using a 2-group test with a .05 two-sided significance when comparing mean changes in primary outcome between study arms. We anticipate a relatively larger effect size because the study time point is in a shorter time frame after treatment. The effect size estimated from this pilot study will be used to plan a future full-scale, multiyear trial.

### Ethical Considerations

#### Institutional Review Board Approval

The study procedures were reviewed and approved by the VA Greater Los Angeles (VAGLA) institutional review board (IRB; approval 1754210) and University of California, Los Angeles, and the full trial was registered through ClinicalTrials.gov (NCT06039865). Any modifications to the protocol related to deviation of study aim, study design, patient populations, or study procedures will not be implemented until the formal amendment to the protocol is approved by the IRB.

#### Privacy and Informed Consent

A Health Insurance Portability and Accountability Act waiver will be requested for the chart review. Patients who are eligible based on preliminary telephone screening will be invited to participate in an informed consent appointment where the risks and benefits of the study will be presented. After written informed consent (either paper or electronic) is obtained, participants will undergo a baseline assessment. Data will be deidentified.

### Data Management

Electronic data will be stored behind a VA firewall on a secure research server, with access limited to the research team. Paper charts will be secured in locked filing cabinets in locked research offices with access limited to the research team. PAP modem reports will be downloaded and data extracted and converted to data files using an automated process. Random files (10%) will be manually extracted, and these manually extracted data will be compared to the automated process. Data will be cleaned over the course of the study. All personal information about potential and enrolled participants will only be accessible to study team members before, during, and after the trial to protect confidentiality. We will retain and dispose of records in compliance with VA Record Control Schedules [20].

### Monitoring and Auditing

A data and safety monitoring board will not be needed for this study because our study is a single-site clinical trial involving the use of PAP machines that is considered standard of care for patients with OSA. We will be pairing the use of PAP machines with a consumer-grade wearable device but using both together should pose minimal risk to the patient because none of the data collected will be used for diagnosing purposes. We will conduct a check-in visit with patients to ask about their experience with the program, which will include questions about adverse events related to study participation. We will follow VA reporting requirements to our IRB for adverse events and serious adverse events.

VAGLA Compliance conducts monthly audits to ensure compliance with procedures for informed consent. This process is independent from investigators.

## Results

Amendments were approved by VAGLA IRB in October 2023 and in March 2024. Primary reason for the March 2024 protocol amendment was as follows: protocol updates included addition of nonadherence to PAP therapy (use of PAP device for 4 or more hours per night on 70% of nights during a consecutive 30-day period) as inclusion criteria; changed collection of 3 nights oximetry at baseline assessment to 1 night of oximetry if no prior overnight oximetry results available; added PROMIS Sleep Disturbance Short Form and Sleep-Related Impairment Short Form and removed Pittsburg Sleep Quality Index and Insomnia Severity Index as baseline assessment instruments; and clarified that Symptoms, Tiredness, Alertness, Mood, and Psychosocial, PROMIS Sleep Disturbance Short Form, Sleep-Related Impairment Short Form, and determinants of nasal continuous PAP compliance would be asked at T3 and T5 and that the Acceptability of Intervention Measures, Intervention Appropriateness Measures, and Feasibility of Intervention Measures would be collected from participants after they complete the intervention program. Primary reason for A2 protocol amendment included protocol updated by adding VA DocuSign as a method of obtaining written consent during VA telehealth consent visit.

Recruitment began in October 2023 and should be completed by September 2024. Results will be disseminated through conferences and peer-reviewed journals in the sleep, digital health, and health services fields.

## Discussion

### Anticipated Findings

The proposed pilot study will examine the impact and acceptability of augmenting current PAP therapy data with a consumer-grade wearable device to promote self-management of PAP therapy for OSA. Using SpO_2_ data collected by a consumer-grade wearable represents an opportunity to support self-management of OSA to increase the use of PAP therapy and promote dialogue between patients and their sleep clinicians about OSA treatment. This pilot trial will provide important information regarding the acceptability of the consumer wearable intervention.

Underuse of PAP therapy is common among patients with OSA. Improving adherence to PAP therapy remains a formidable task, and new approaches that can be used alone or in combination with existing programs to improve adherence are needed. The growing use of consumer wearables provides a new opportunity to increase and optimize the use of PAP therapy. Including a consumer-grade wearable device to collect pulse oximetry data nightly and discussing the data presented in a customized report with a clinician as part of the patient’s OSA therapy represents a new approach. The self-monitoring program provides participants with the support they might need between clinic visits, agency to manage their own treatment, and self-awareness. The intervention was designed to maximize accessibility by incorporating internet-based modalities such as e-surveys and video visits to allow participants who live further away from the sleep clinic to participate in the study, thus ensuring access to care and tools for these patients who may experience greater barriers to attending follow-up visits.

### Limitations

There may be data limitations to using pulse oximetry data collected by a consumer-grade wearable device. We may encounter instances where there are missing data from part of the night when a participant was asleep, which could be due to the position of the wearable while sleeping. Anticipating this issue, we have established a procedure to notify the participant that no data had been collected for the previous night and to remind them to wear the device on their nondominant wrist. In addition, we do make it clear that the report will be based on available data only, which is adequate for the purposes of self-monitoring. There may also be situations where the smartwatch fails to sync and does not transmit to the third-party platform for the research team to download. For these instances, we will check syncing weekly and call participants who do not have a sync in the past 24 hours.

As our inclusion criteria will allow for either use of Respironics or ResMed device, there could be potential discrepancies in PAP use reports due to manufacturer algorithm differences. A study by Nolan et al [35] compared adherence to an older Respironics versus older ResMed device versus Breas and found no significant difference between the Respironics versus ResMed device (there was significant difference for the Breas, but our sleep center has never used Breas). Although Isetta et al [36,37] reported differences in the algorithms between Respironics and ResMed device, noncompliance is multifactorial such that the device algorithm could be just one factor among many other non–pressure-related factors. Moreover, larger studies comparing fixed versus flexible pressure settings identified no difference in adherence for new users [7]. We will attempt to perform stratified subanalyses for patients prescribed ResMed versus Respironics devices if we end up enrolling participants using CPAP devices by different manufacturers.

Some participants may have difficulty understanding and applying information from the charts [38]. To increase the utility of the weekly report displaying nightly oxygen saturation data and patient-reported outcomes on sleep quality, mood, and functioning, our study will include a check-in video visit on day 7 to orient the participant to the weekly report, which may promote participant engagement [39]. We will explore the effect of these sessions as well as participant experience with the intervention with semistructured interviews conducted at the end of the research study.

Some studies have suggested that knowing there will be a delay in receiving the intervention may influence a participant’s behavior such that it is no longer an accurate portrayal of their natural behavior while awaiting treatment [40]. Some participants may in fact put less effort in their current treatment, potentially exacerbating their symptoms. This could result in an inflated estimated effect of the intervention if waitlist control participants improve “significantly” once in treatment. However, our trial’s wait time is brief (6 weeks), which is not very different from other delays participants may have encountered in receiving sleep equipment due to recent device recalls [41]. We will not use the term “waitlist,” which may carry negative connotations, for participants who will start the intervention after an initial waiting period.

Finally, we may encounter a selection bias due to participants who may not be comfortable with video visits or using a wearable device declining participation. This is a common issue with trials involving technology. In a future large-scale trial, we could work with patients who decline due to discomfort with technology before randomization and give them more time to acclimate to using video visits or a wearable device.

### Possible Implications

This study will provide an opportunity to explore SpO_2_ as a use case of patient-generated health data for OSA self-management and to assess the effect of sharing these data with patients on PAP adherence. In the proposed trial, nightly SpO_2_ data will be visualized to show the amount of time in normal and abnormal oxygen saturation while sleeping along with sleep-related patient-reported outcomes (eg, daytime sleepiness) over a 7-day period. To our knowledge, SpO_2_ data have not been visualized in this manner.

This intervention will also pair the weekly report with a discussion with a research team member after week 1 so the patient can learn to interpret the figure and apply insights for OSA self-management. Findings from the study may provide additional insights on how patients with OSA might use patient-generated health data collected by consumer wearables to inform self-management of OSA and possibly increase their use of PAP therapy. When patients are provided with meaningful data, in the form of oxygen saturation and their symptoms, and they are able to identify patterns and relationships between these data points, this may help increase patient awareness of their condition and lead to better decision-making about their treatment [42]. We will be examining the utility of the curated data visualizations delivered weekly to further our understanding of how to deploy this type of intervention to improve use of PAP therapy and promote patient engagement in their own care.

Promoting engagement with one’s care is vital to management of OSA. Our study will examine the effect of providing support to patients with OSA with a self-monitoring program between clinic visits. The intervention will provide patients with curated data visualizations and will also assist patients with interpreting these weekly reports. Findings from examining the acceptability and efficacy of augmenting current PAP therapy with wearable oxygen data will enable us to optimize implementation of patient-generated health data as an enhancer of daily living and routine clinic care. Findings will contribute additional knowledge regarding the implementation of interventions aimed at increasing OSA self-management practices.

## Appendix

Multimedia Appendix 1 SPIRIT Checklist.

## Abbreviations

IRB: institutional review board
mPAP: Chronic Care Management With Wearable Devices in Patients Prescribed Positive Airway Pressure Therapy
OSA: obstructive sleep apnea
PAP: positive airway pressure
PROMIS: Patient-Reported Outcomes Measurement Information System
SPIRIT: Standard Protocol Items: Recommendations for Interventional Trials
SpO2: blood oxygen saturation
VA: Veterans Affairs
VAGLA: Veterans Affairs Greater Los Angeles

## Data Availability

The data sets generated during and analyzed during this study are available from the corresponding author on reasonable request.

## References

[1] Young T, Peppard PE, Gottlieb DJ (2002). Epidemiology of obstructive sleep apnea: a population health perspective. Am J Respir Crit Care Med.

[2] Omachi TA, Claman DM, Blanc PD, Eisner MD (2009). Obstructive sleep apnea: a risk factor for work disability. Sleep.

[3] Epstein LJ, Kristo D, Strollo Jr PJ, Friedman N, Malhotra A, Patil SP, Ramar K, Rogers R, Schwab RJ, Weaver EM, Weinstein MD (2009). Clinical guideline for the evaluation, management and long-term care of obstructive sleep apnea in adults. J Clin Sleep Med.

[4] (2023). Positive Airway Pressure (PAP) devices for the treatment of obstructive sleep apnea. Medicare Coverage Database.

[5] (2021). Local coverage determination (LCD) for positive airway pressure (PAP) devices for the treatment of obstructive sleep apnea (L11518). Medicare Coverage Database.

[6] Timkova V, Nagyova I, Reijneveld SA, Tkacova R, van Dijk JP, Bültmann U (2020). Quality of life of obstructive sleep apnoea patients receiving continuous positive airway pressure treatment: a systematic review and meta-analysis. Heart Lung.

[7] Sawyer AM, Gooneratne NS, Marcus CL, Ofer D, Richards KC, Weaver TE (2011). A systematic review of CPAP adherence across age groups: clinical and empiric insights for developing CPAP adherence interventions. Sleep Med Rev.

[8] Liang Z, Ploderer B, Liu W, Nagata Y, Bailey J, Kulik L, Li Y (2016). SleepExplorer: a visualization tool to make sense of correlations between personal sleep data and contextual factors. Pers Ubiquit Comput.

[9] Park S, Bekemeier B, Flaxman A, Schultz M (2021). Impact of data visualization on decision-making and its implications for public health practice: a systematic literature review. Inform Health Soc Care.

[10] Samsung’s sleep apnea feature on Galaxy watch first of its kind authorized by US FDA. Samsung.

[11] Birrer V, Elgendi M, Lambercy O, Menon C (2024). Evaluating reliability in wearable devices for sleep staging. NPJ Digit Med.

[12] Kainec KA, Caccavaro J, Barnes M, Hoff C, Berlin A, Spencer RM (2024). Evaluating accuracy in five commercial sleep-tracking devices compared to research-grade actigraphy and polysomnography. Sensors (Basel).

[13] Bianchi MT (2018). Sleep devices: wearables and nearables, informational and interventional, consumer and clinical. Metabolism.

[14] Chinoy ED, Cuellar JA, Huwa KE, Jameson JT, Watson CH, Bessman SC, Hirsch DA, Cooper AD, Drummond SP, Markwald RR (2021). Performance of seven consumer sleep-tracking devices compared with polysomnography. Sleep.

[15] Feng S, Mäntymäki M, Dhir A, Salmela H (2021). How self-tracking and the quantified self promote health and well-being: systematic review. J Med Internet Res.

[16] Rafl J, Bachman TE, Rafl-Huttova V, Walzel S, Rozanek M (2022). Commercial smartwatch with pulse oximeter detects short-time hypoxemia as well as standard medical-grade device: validation study. Digit Health.

[17] Hwang D, Chang JW, Benjafield AV, Crocker ME, Kelly C, Becker KA, Kim JB, Woodrum RR, Liang J, Derose SF (2018). Effect of telemedicine education and telemonitoring on continuous positive airway pressure adherence. The Tele-OSA randomized trial. Am J Respir Crit Care Med.

[18] Becker MH (1974). The health belief model and sick role behavior. Health Educ Monogr.

[19] Chan AW, Tetzlaff JM, Altman DG, Laupacis A, Gøtzsche PC, Krleža-Jerić K, Hróbjartsson A, Mann H, Dickersin K, Berlin JA, Doré CJ, Parulekar WR, Summerskill WS, Groves T, Schulz KF, Sox HC, Rockhold FW, Rennie D, Moher D (2013). SPIRIT 2013 statement: defining standard protocol items for clinical trials. Ann Intern Med.

[20] Naik S, Al-Halawani M, Kreinin I, Kryger M (2019). Centers for Medicare and Medicaid services positive airway pressure adherence criteria may limit treatment to many Medicare beneficiaries. J Clin Sleep Med.

[21] Stepnowsky Jr CJ, Marler MR, Ancoli-Israel S (2002). Determinants of nasal CPAP compliance. Sleep Med.

[22] Case study: responsible AI: the Fitbit blood oxygen saturation (SpO2) tracking feature. Google AI.

[23] Selim AJ, Fincke G, Ren XS, Lee A, Rogers WH, Miller DR, Skinner KM, Linzer M, Kazis LE (2004). Comorbidity assessments based on patient report: results from the Veterans Health Study. J Ambul Care Manage.

[24] Weaver TE, Maislin G, Dinges DF, Bloxham T, George CF, Greenberg H, Kader G, Mahowald M, Younger J, Pack AI (2007). Relationship between hours of CPAP use and achieving normal levels of sleepiness and daily functioning. Sleep.

[25] Weiner BJ, Lewis CC, Stanick C, Powell BJ, Dorsey CN, Clary AS, Boynton MH, Halko H (2017). Psychometric assessment of three newly developed implementation outcome measures. Implement Sci.

[26] Yu L, Buysse DJ, Germain A, Moul DE, Stover A, Dodds NE, Johnston KL, Pilkonis PA (2011). Development of short forms from the PROMIS™ sleep disturbance and sleep-related impairment item banks. Behav Sleep Med.

[27] Jones J, Nielson SA, Trout J, Swenson M, Reiley J, Tanner J, Bowers D, Kay DB (2021). A validation study of PROMIS sleep disturbance (PROMIS-SD) and sleep related impairment (PROMIS-SRI) item banks in individuals with idiopathic Parkinson’s disease and matched controls. J Parkinsons Dis.

[28] Johns MW (1991). A new method for measuring daytime sleepiness: the Epworth sleepiness scale. Sleep.

[29] Gonçalves MT, Malafaia S, Moutinho Dos Santos J, Roth T, Marques DR (2023). Epworth sleepiness scale: a meta-analytic study on the internal consistency. Sleep Med.

[30] Okan Y, Janssen E, Galesic M, Waters EA (2019). Using the short graph literacy scale to predict precursors of health behavior change. Med Decis Making.

[31] Mehta N, Mandavia R, Patel A, Zhang H, Liu Z, Kotecha B, Veer V (2020). Patient-reported outcome measure for obstructive sleep apnea: symptoms, tiredness, alertness, mood and psychosocial questionnaire: preliminary results. J Sleep Res.

[32] Belin TR, Gjertson DW, Hu MY (1997). Summarizing DNA evidence when relatives are possible suspects. J Am Stat Assoc.

[33] Liu MT, Taylor JM, Belin TR (2000). Multiple imputation and posterior simulation for multivariate missing data in longitudinal studies. Biometrics.

[34] Schafer JL (1997). Analysis of Incomplete Multivariate Data.

[35] Nolan GM, Ryan S, O'connor TM, McNicholas WT (2006). Comparison of three auto-adjusting positive pressure devices in patients with sleep apnoea. Eur Respir J.

[36] Isetta V, Navajas D, Montserrat JM, Farré R (2015). Comparative assessment of several automatic CPAP devices' responses: a bench test study. ERJ Open Res.

[37] Isetta V, Montserrat JM, Santano R, Wimms AJ, Ramanan D, Woehrle H, Navajas D, Farré R (2016). Novel approach to simulate sleep apnea patients for evaluating positive pressure therapy devices. PLoS One.

[38] Grossman LV, Feiner SK, Mitchell EG, Masterson Creber RM (2018). Leveraging patient-reported outcomes using data visualization. Appl Clin Inform.

[39] Coşkun A, Karahanoğlu A (2022). Data sensemaking in self-tracking: towards a new generation of self-tracking tools. Int J Hum Comput Interact.

[40] Mohr DC, Spring B, Freedland KE, Beckner V, Arean P, Hollon SD, Ockene J, Kaplan R (2009). The selection and design of control conditions for randomized controlled trials of psychological interventions. Psychother Psychosom.

[41] Owens RL, Wilson KC, Gurubhagavatula I, Mehra R (2021). Philips respironics recall of positive airway pressure and noninvasive ventilation devices: a brief statement to inform response efforts and identify key steps forward. Am J Respir Crit Care Med.

[42] Ravichandran RS, Patel SW, Kientz JA, Pina LR (2017). Making sense of sleep sensors: how sleep sensing technologies support and undermine sleep health. Proceedings of the 2017 CHI Conference on Human Factors in Computing Systems.

